# Medication Adherence and Quality of Life in Epilepsy: The Potential Role of Seizure Severity in the Association Between Them

**DOI:** 10.3390/jcm15093311

**Published:** 2026-04-27

**Authors:** Nurlybek Mombekov, Nigara Yerkhojayeva, Islamkhan Doszhanov, Nazira Zharkinbekova, Gulnaz Nuskabayeva, Karlygash Sadykova, Assylbek Mombek, Sandugash Rustemova, Aigerim Togizbayeva, Nursultan Nurdinov

**Affiliations:** 1 Department of Special Clinical Disciplines, Faculty of Medicine, Khoja Akhmet Yassawi International Kazakh-Turkish University, Turkestan 161200, Kazakhstan; 2 Department of Neurology, Psychiatry, Rehabilitation and Neurosurgery, South Kazakhstan Medical Academy, Shymkent 160019, Kazakhstan; 3 Department of Fundamental Medical Sciences, Faculty of Dentistry, Khoja Akhmet Yassawi International Kazakh-Turkish University, Turkestan 161200, Kazakhstan

**Keywords:** epilepsy, medication adherence, seizure severity, quality of life, cognitive impairment, Kazakhstan

## Abstract

**Background/Objectives:** Epilepsy is a long-term condition that affects the brain and has a big impact on a person’s daily life, especially in areas where people do not have a lot of money or access to good healthcare. This study aimed to evaluate the relationship between medication adherence and QoL and to assess the role of seizure severity in the association between them among patients with epilepsy. **Methods:** A cross-sectional study of 1100 adult patients with epilepsy was conducted using registry data and structured interviews. The main outcomes that were assessed are quality of life (QoL), medication adherence, and seizure severity. **Results:** Reduced QoL was observed in 62% of patients. Low medication adherence was significantly associated with reduced QoL (OR = 4.33 [3.24–5.79] unadjusted; 3.90 [3.07–5.80] fully adjusted). Seizure severity was also associated with reduced QoL (OR = 1.62, *p* = 0.002; OR = 2.05, *p* < 0.001). Cognitive impairment showed the strongest association with reduced QoL, with ORs of 14.6 for mild and 80.8 for moderate-severe impairment in unadjusted models, remaining significant after adjustment. Medication adherence was significantly associated with seizure severity (OR = 1.18, *p* = 0.002), and attenuation of its effect after adjustment suggests that these variables are interrelated, although causality cannot be determined in this study. Additional factors associated with reduced QoL included lower education, longer disease duration, polytherapy, structural brain abnormalities, and comorbidities. **Conclusions:** Reduced QoL in epilepsy is strongly influenced by cognitive impairment and medication nonadherence, with seizure severity potentially contributing to this association, although causality cannot be inferred. These findings support integrated care strategies targeting adherence, cognition, and seizure control to improve patient outcomes.

## 1. Introduction

Epilepsy, a chronic disorder characterized by recurrent unprovoked seizures, is a global public health problem affecting about 50–65 million individuals in the world [1]. Higher prevalence of the condition has been reported from low- and middle-income countries, due to limited availability of tools for accurate diagnosis and optimal management of the condition. Moreover, inadequate management of the condition also imposes huge economic and psychosocial burdens, in particular in resource-poor settings [2]. The first-line management of the condition is with antiseizure medications (ASMs) with the expectation that optimal therapy achieved through adherence to medication will achieve seizure control in 60–70% of patients with epilepsy [3]. Despite their efficacy, medication nonadherence, however, remains the commonest problem in the management of epilepsy and there is considerable interindividual and also between different populations of patients with epilepsy in the degree of adherence to the therapeutic regimen achieved, ranging from about 30% to 70% [4].

Poor adherence may influence uncontrolled seizures, as the seizures become more frequent and severe, affecting quality of life (QoL) [5]. QoL in epilepsy is composed of physical, psychological, social, and other factors such as limitations due to postictal fatigue, mental and behavior changes including anxiety and depression, cognitive deficits, and social restrictions. Cross-sectional and longitudinal studies have shown that good adherence to ASMs is associated with higher QoL, but the factors influencing this relationship have not been fully investigated [6]. Recently, it was found that seizure severity may play an important role in this relationship [5,7]; previous studies suggest complex, potentially bidirectional relationships between adherence, seizure control, and QoL, although the direction of these associations remains uncertain.

Despite the growing body of knowledge on factors influencing treatment outcomes in epilepsy, there is still a paucity of information especially in non-Western populations in whom cultural beliefs about epilepsy and healthcare access may pose a significant barrier to good adherence to ASMs. There is a particular lack of data from Central Asia, including Kazakhstan, where healthcare systems face challenges typical of lower- and middle-income countries (LMICs), such as limited access to specialized care and variability in treatment continuity. This study will investigate the role of seizure severity in the relationship between AED adherence and health-related quality of life in Kazakhstan.

## 2. Materials and Methods

### 2.1. Study Design and Population

This cross-sectional study included adult patients with epilepsy who were recorded in the Turkestan or Kyzylorda (South Kazakhstan) regional epilepsy registry. Patients who were included in the study were between 18 and 75 years of age, and had a definite clinical diagnosis of epilepsy and a duration of two years or longer. All the patients were receiving a current neurological treatment and gave consent to participate in the study. Eligible patients were excluded from the study if their medical condition required immediate attention in the hospital because of non-seizure, systemic medical concerns, had inadequate seizure documentation, or had significant clinical deterioration. Patients were also excluded if they had a prior, severe drug reaction to AEDs or a history of alcohol or substance abuse.

### 2.2. Exposures and Covariates

All relevant data were obtained from the patients’ medical records and from a structured patient interview. Sociodemographic information consisted of age, gender, ethnicity, marital status, highest level of education, main occupation, and their residential area. The following clinical variables were recorded: age at onset, disease duration, seizure type, seizure frequency, time to occur, presence of sequential seizures or status epilepticus, number of administered ASMs, family history of seizures, postictal confusion, and presence of other concurrent medical conditions. Computed tomography or magnetic resonance imaging (CT/MRI) neurophysiological and neuroimaging data were categorized as normal or abnormal.

### 2.3. Assessed Measurements

Quality of life was evaluated using the Quality of Life in Epilepsy questionnaire-31 items (QOLIE-31), a valid assessment tool that examines various aspects of life such as mental health, social behavior, and issues related to seizures [8]. The total score is from 0 to 100; and scores were categorized as good (67–100) and moderately or severely reduced (0–66) for analysis. Medication adherence was assessed by using the 8-item Morisky Medication Adherence Scale (MMAS-8), a widely used self-reporting tool developed for evaluation of adherence-related behaviors including forgetting to take medications and intentionally not taking them [9]. Scores were divided into three levels as low (<6), medium (6 to <8), and high (8 points).

Seizure severity was evaluated using the Liverpool Seizure Severity Scale (LSSS), a clinical scale designed to assess the impact of a seizure on the individual and the consequences of seizures [10]. The LSSS scores were rated as mild (1–15 points), moderate (16–30 points), or severe (31 or more). The Montreal Cognitive Assessment (MoCA) was used to assess cognitive function. It is a tool designed to screen for cognitive impairment in various fields, including attention, memory, and executive functions [11]. MoCA scores were categorized into normal (≥26), mild cognitive impairment (18–25), and moderate to severe cognitive impairment (<18). Stigma was measured using the Epilepsy Stigma Scale (ESS), which assesses the stigmatization experienced by patients [12]. ESS scores were categorized as low (10–23 points), average (24–46 points), and high (47–70 points).

All the questionnaires (QOLIE-31, MMAS-8, LSSS, MoCA, and ESS) were translated into the Russian and Kazakh languages using the forward-backward translation procedure. Validation of the translated versions was confirmed by pilot-testing them in a separate group of 50 patients with epilepsy excluded from the main analysis. While this process supported face validity and feasibility of the instruments in the study setting, formal psychometric validation (e.g., reliability testing or construct validity assessment) was not performed as part of this study.

### 2.4. Statistical Analysis

Descriptive statistics were used to summarize the study population. Continuous variables were presented as means with standard deviations (SD), and categorical variables as frequencies and percentages. Differences between QoL groups were assessed using Student’s *t*-test for normally distributed continuous variables and the Mann–Whitney U test for non-normally distributed variables. Categorical variables were compared using the χ^2^ test or Fisher’s exact test, as appropriate. For ease of presentation and interpretation, all scale-based variables (QOLIE-31, MMAS-8, LSSS, MoCA, and ESS) were analyzed in their categorized forms rather than as continuous measures.

Multivariable logistic regression models were used to evaluate factors associated with reduced QoL. The following three models were constructed: an unadjusted model, Model 1 adjusted for all covariates except seizure severity, and Model 2 fully adjusted including seizure severity. Results were reported as odds ratios (ORs) with 95% confidence intervals (CIs). A reduction in the effect estimate for medication adherence after inclusion of seizure severity was interpreted as statistical attenuation after adjustment, without implying causal pathways. Model fit was assessed using the Hosmer–Lemeshow test and model discrimination using the area under the ROC curve (AUC). Multicollinearity was evaluated using variance inflation factors (VIF). All analyses were conducted using Stata (version 16, StataCorp LLC, College Station, TX, USA), with a two-sided *p*-value < 0.05 considered statistically significant.

## 3. Results

Of 1100 people with epilepsy, 419 (38%) had a good quality of life, while 681 (62%) had moderately or severely reduced QoL (Table 1). Patients with reduced QoL had a significantly higher age at epilepsy onset compared to those with good QoL (28 vs. 25 years of age, *p* < 0.001). Sex distribution and place of residence were comparable between groups and not associated with QoL.

Education level was significantly associated with QoL (*p* < 0.001), with higher education more frequent among patients with good QoL and lower education more prevalent in those with reduced QoL. Marital status also differed between groups (*p* < 0.001), as follows: married individuals were more likely to report good QoL, whereas widowed patients were overrepresented in the reduced QoL group (Table 1). According to the results of this study, patients who do not have a disability tend to have a better QoL, whereas those with more severe disabilities, especially those in Group II, are more likely to experience a reduced QoL (*p* < 0.001). Additionally, income level also plays a role in determining QoL, with noticeable differences across various income categories.

Table 2 represents clinical characteristics of patients with epilepsy. The mean duration of epilepsy was longer in poor quality of life than in good QoL (15 ± 10 years vs. 11 ± 8 years, *p* < 0.001. Moderately or severely reduced QoL was associated with the use of polytherapy and with a higher frequency of administration of drugs (*p* = 0.006 and *p* = 0.007, respectively). Patients with structural brain abnormalities had a higher frequency of lower scores, while patients with normal MRI findings had higher QoL scores (84% vs. 75%, *p* < 0.001). Comorbidities were also found to be significantly associated with QoL (*p* < 0.001). The lower QoL group had a higher prevalence of mental and neurological disorders. Drug-resistance was significantly associated with reduced QoL, while family history of epilepsy did not show association with quality of life.

Table 3 presents seizure-related characteristics of patients. Epilepsy of structural etiology was seen more frequently in patients with lower quality of life, while epilepsy of unknown etiology was higher in patients with higher quality of life (*p* < 0.001). Patients with lower QoL more often had generalized seizures and secondary generalized seizures, higher seizure frequency, postictal confusion, and serial seizures (all *p* < 0.001). Seizure time did not have significant association with QoL. The distribution of aura types differed significantly between groups (*p* < 0.001), with somatosensory and abdominal auras being the most common, and slightly higher proportions of auditory, emotional, and mental auras observed among patients with reduced QoL.

The post-seizure state was strongly associated with quality of life (*p* < 0.001), and there were more adverse postictal effects in the group with lower QoL. The neurological examination was highly correlated with quality of life (*p* < 0.001). Normal examination results were more frequently recorded in patients with good quality of life, and focal neurological signs were more frequently observed in patients with decreased quality of life. Provoking factors differed significantly between groups (*p* = 0.003), with reflex triggers being the most common overall, while alcohol consumption, treatment withdrawal, and lack of sleep were more frequently reported among patients with reduced QoL.

As shown in Table 4, low medical adherence was markedly more common among patients with reduced QoL (86% vs. 58%), while medium adherence was more frequent in those with good QoL (41% vs. 14%) (*p* < 0.001). Low adherence to the medication was seen more frequently in patients with poor quality of life (86% vs. 58%), and medium adherence to the medication was seen more frequently in patients with good quality of life (41% vs. 14%). Patients with poor quality of life had moderate to severe seizures, while those with good QoL had mild seizures (*p* = 0.004).

The frequency of moderate to severe cognitive impairment was higher in the reduced QoL group (22% vs. 1%) than in the preserved QoL group, while normal cognitive function was more frequent in the good QoL group (10% vs. 1%), with *p* < 0.001. A high stigma score was more often observed among those with a lower quality of life (31% vs. 5%), whereas a low stigma score was more often observed among those with a good QoL (30% vs. 3%), with *p* < 0.001.

**Figure 1:**
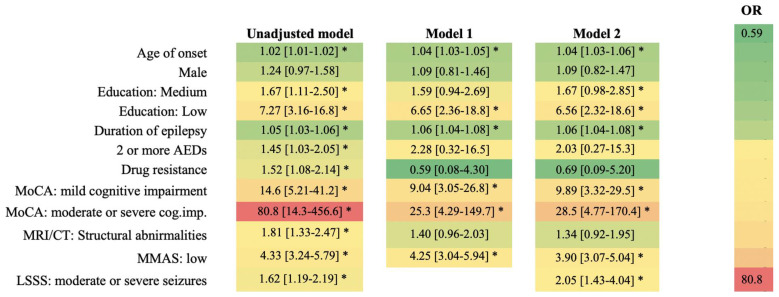
Heatmap of unadjusted and adjusted associations between clinical and patient-related factors and reduced quality of life in patients with epilepsy. * *p* < 0.05 (statistically significant).

The heatmap in Figure 1 shows that cognitive impairment has the biggest impact on reducing quality of life, across all the different models. In an unadjusted model, people with mild cognitive impairment and moderate or severe impairment have 14 times and 80 times, respectively, higher odds of lower quality of life (*p* < 0.001). Even after adjustment on other factors, the connection between cognitive impairment and lower quality of life remains strong and statistically significant (Figure 1).

The severity of seizures was linked to a lower quality of life (OR_unadj_ = 1.62 [1.19–2.19], *p* = 0.002 and OR_adj_ = 2.05 [1.43–2.04], *p* < 0.001). Low medication adherence was consistently associated with reduced QoL, with OR = 4.33 [3.24–5.79] in the unadjusted model, OR = 4.25 [3.04–5.04] in Model 1, and OR = 3.90 [3.07–5.80] in Model 2. Moreover, medication adherence was also closely tied to the severity of their seizures, with OR_adj_ = 1.18 [1.01–1.48], *p* = 0.002, as seen in Supplementary Table S1. The attenuation of the MMAS effect in Model 2 suggests that seizure severity is an important correlated factor that influences the observed association between adherence and QoL. The wide confidence intervals observed for some variables in the seizure severity model suggest limited model stability, likely due to small subgroup sizes. Therefore, these results should be interpreted cautiously, and the model should not be used to support mechanistic or pathway-based interpretation.

To assess the robustness of these findings, additional analyses were performed using continuous representations of MoCA, MMAS, and LSSS scores (Supplementary Table S2). In these models, the direction of associations remained consistent. Higher MoCA scores were associated with lower odds of reduced QoL (OR = 0.63 [0.58–0.68], *p* < 0.001), while higher LSSS scores were associated with increased odds of reduced QoL (OR = 1.04 [1.01–1.08], *p* = 0.011). Similarly, higher MMAS scores were associated with lower odds of reduced QoL (OR = 0.55 [0.46–0.65], *p* < 0.001).

## 4. Discussion

This study demonstrated that reduced quality of life is highly prevalent among patients with epilepsy in Southern Kazakhstan, affecting nearly two-thirds of participants. Cognitive impairment and poor medication adherence emerged as the strongest factors related to reduced QoL, while seizure severity was significantly associated with QoL and appeared to partially explain the relationship between adherence and QoL. These findings highlight the multifactorial nature of QoL in epilepsy, encompassing both clinical and psychosocial dimensions. Importantly, these findings provide insights into patients with established epilepsy receiving care in registry-based settings in Kazakhstan, where structural factors such as access to specialized care, availability of medications, and continuity of follow-up may influence both clinical outcomes and quality of life.

### 4.1. Cognitive Impairment and QoL

Cognitive impairment showed the strongest association with reduced QoL in all models. However, this finding should be interpreted with caution. The QOLIE-31 includes domains related to cognitive functioning, and the use of MoCA as a predictor introduces conceptual overlap between the exposure and outcome measures. This overlap may partly contribute to the magnitude of the observed associations, particularly the very large odds ratios for moderate-to-severe cognitive impairment. Similarly, seizure severity as measured by the LSSS captures the impact and consequences of seizures, which conceptually overlap with QoL domains assessed by the QOLIE-31. Therefore, part of the observed association between seizure severity and QoL may reflect shared constructs between these instruments rather than entirely independent effects. Several studies conducted in Germany [1], Slovenia [2], North America [3,4], and Iran [5] have demonstrated the great correlation between cognitive functions—attention, memory, and executive functions—and the degree of reduced level of daily activities, autonomy, and social relationships that people with epilepsy experience. Other multicenter studies have also already proved that the cognitive impairment often has greater influence on the quality of life of people with epilepsy than clinical factors such as the frequency or type of seizures [6].

Several hypotheses have been proposed in the literature regarding the etiology of this association including the effects of recurrent seizures [7], underlying neuropsychological deficits [8], and long-term cognitive effects of chronic antiseizure medication use, particularly when used in a polytherapy regimen [9,10]. However, the observed association between polytherapy and reduced QoL should be interpreted with caution, as it may reflect confounding by indication. Patients receiving multiple ASMs are more likely to have more severe or refractory epilepsy, which itself is associated with poorer outcomes. Additionally, social issues may impact the quality of life of individuals with epilepsy such as social stigma, reduced educational and employment opportunities, and hopelessness. Moreover, the strong association between cognitive impairment and low QoL in this study may be as a result of under-diagnosis and poor management of cognitive impairment in our study setting. In fact, similar observations have been reported in other low- and middle-income countries where specialized cognitive assessments and rehabilitation services are lacking to meet the unmet cognitive and psychosocial needs of people with epilepsy [11]. Cognitive impairment may be influenced both by the underlying neurological condition and by treatment-related factors, including the use of multiple ASMs.

According to the results of this study, patients with a decreased QoL had a higher age at epilepsy onset. Several explanations may underlie this finding, including the possibility that different etiologies are responsible. Late-onset epilepsy is often caused by structural factors and typically carries a higher neurological burden of disease with associated comorbid conditions [12,13]. Patients who develop epilepsy later in life may also find it more difficult to deal with as a result of their current social role, occupation, and way of life [14]. Furthermore, the potential for neuroplastic changes may be limited in such patients. Control of seizures is still the cornerstone of the treatment of patients with epilepsy. In light of the present study, we propose that the standard care of patients with epilepsy should be modified in order to be more patient oriented, and that cognitive function should be assessed regularly. Early recognition of cognitive impairments in epilepsy enables targeted interventions like cognitive therapy, medication adjustments, and performance adaptations, ultimately enhancing long-term management and quality of life [15,16].

### 4.2. Medication Adherence and Its Role

Low medication adherence was consistently related to higher odds of poor quality of life, according to the results of the current study. After adjustment for potential confounding variables, nonadherence to AEDs was associated with poor seizure control as reported in other international studies investigating the effects of nonadherence to AEDs [17,18]. The issue of adherence to therapy is multifaceted. It is not just about having appropriate drug exposure, but it is also about the degree to which a patient engages in the treatment regimen, health literacy, and the patient’s beliefs regarding their disease and its management. For instance, patients who have concerns about long-term medication effects or who lack adequate understanding of epilepsy management are more likely to demonstrate inconsistent medication use, which in turn negatively affects both clinical outcomes and QoL [19,20].

The strength of association observed in our study is comparable or even greater than that found in studies from high-income settings. It is possible that in settings where the infrastructure to support adherence such as adherence support programs, mobile phone-based monitoring, and health education is not developed, the nonadherence to treatment may have severe consequences [21,22]. This has also been observed in other low- and middle-income countries, where nonadherence was attributed to a number of barriers including the cost of the drugs, access to the health care services, and irregular drug supply, and these barriers have been associated with a poor quality of life [23].

Adherence and quality of life are likely related in a complex, multifaceted manner, with adherence having a direct and indirect impact on quality of life. In LMIC settings, including Kazakhstan, barriers such as medication cost, irregular drug supply, and limited healthcare access may further amplify the impact of nonadherence on patient outcomes [24]. Nonadherence may contribute to inadequate seizure control and through a complex interplay with other variables such as depression, anxiety, and cognitive function affect the overall quality of life [18,25]. Thus, improving the management of nonadherence in people with epilepsy will be valuable. Therefore, patient education, simple dosing regimens, and adherence-enhancing strategies are straightforward and inexpensive interventions that are easy to implement as part of the standard care provided to individuals with epilepsy and can therefore be easily beneficial to both the physician and the patient.

### 4.3. Association of Seizure Severity, Medication Adherence, and QoL

In this study, seizure severity was independently associated with quality of life and influenced the magnitude of the association between medication adherence and QoL after adjustment. However, this attenuation should be interpreted cautiously without causality due to the cross-sectional design of the study. The adherence effect was reduced after adjusting for seizure severity, and lower adherence was associated with greater seizure severity, which also suggests that these factors are interrelated. This result is consistent with a large body of international literature showing that seizure burden in terms of seizure severity and unpredictability is one of the most powerful clinical factors predicting poor QoL in people with epilepsy, irrespective of seizure type [26,27].

Other studies in both high [28,29] and low resource settings [30,31] have shown that nonadherence to AED treatment is also strongly associated with higher seizure frequency and severity that associated to the person’s reduced activity level, increased risk of falls and potential injury, and negative psychosocial effects. Our results are consistent with worldwide literature. However, our findings also suggest that adherence to treatment has direct impacts on health-related quality of life independent of seizure severity, effects that have been reported previously in other studies [32,33].

The relationship between adherence, seizure severity, and QoL is likely complex and potentially bidirectional. Our results are consistent with the consensus view based on a broader review of the international literature that adherence management should not be restricted to just reducing seizures, and hence the frequency of seizures as the main endpoint of therapy and should be supplemented with programs to directly address the psychological and social components of nonadherence [20,21].

### 4.4. Seizure-Related Characteristics

According to the results of this study, seizure frequency, seizure severity, type of seizure, postictal confusion, and serial seizures had a negative impact on the QoL, which is consistent with other research literature. The seizure burden is consistently reported as the most significant factor affecting poor quality of life. The present study supports the findings of large multicenter and population-based studies, which also reported that poor seizure control and severe seizures have a profound impact on physical, social, and psychological functioning and that this impact may even surpass that of other clinical factors [34,35].

Generalized seizures are more likely to cause significant impairment due to loss of consciousness, increased risk of injury, and their visibility, which may contribute to stigma [36]. Postictal confusion secondary to serial seizures also results in a longer period of confusion/recovery, greater cognitive disruption, and a greater need for care from others [37], all of which are factors in the impact on the individual’s level of functioning.

Previous studies have shown that the perceived stigma is a major psychosocial challenge to individuals with epilepsy, in both high- [38,39] and low-resource [40] settings. Higher levels of stigma can be associated with poorer social relationships, reduced self-esteem, and noncompliance with medical treatment, all of which are factors in reduced QoL.

### 4.5. Strengths and Limitations

This study has a number of strengths. First, the sample was large and drawn from regional epilepsy registries, which is an advantage of this study within the given context. Second, the instruments used to measure quality of life, adherence, and seizure severity and cognitive function have been validated, which allows for possible comparison with other studies conducted in international settings. Third, the large number of clinical and psychosocial variables that were examined in this study are in line with the modern multidimensional models of epilepsy outcomes, and thus a larger number of factors were considered in the analysis. Finally, the multivariable regression analyses used in this study provide a more sophisticated and rigorous analysis of the associations between the variables under consideration, while allowing for control of the potential confounding effects of other variables and the examination of direct and indirect associations.

However, there are some limitations that should be noted. The cross-sectional design does not allow one to establish causal relationships, nor to determine the temporal relationship of the variables included in the analysis. In this way, poor adherence to the treatment is associated with a lower quality of life, but it is also possible that, in turn, a lower quality of life is associated with poor adherence to the treatment, which could be verified with a better design, such as a prospective design, that allows one to study the variables in their temporal development in time. A second limitation of the study is that adherence was assessed using a self-report questionnaire that is subject to recall and social desirability biases. It is possible that patients over-reported their adherence to their prescribed medications. This could result in the misclassification of the level of adherence to medications and, as a result, any association with outcomes may be attenuated. Thirdly, some degree of residual confounding is inevitable, despite adjustment for potential confounding variables. Unrecorded variables that may be of importance include socioeconomic variables, healthcare activity, patient or family attitudes to epilepsy, and the nature of the AED regime.

The generalizability of the findings is limited by the study population, which was drawn from two regional epilepsy registries and included only adult patients with established epilepsy receiving ongoing treatment. Therefore, the results may not be applicable to newly diagnosed patients, untreated individuals, patients outside registry systems, or those with acute or unstable clinical conditions. Another important limitation is the potential conceptual overlap between the outcome and some of the key predictors, particularly seizure severity and cognitive function. Since QOLIE-31 includes domains related to cognitive functioning and seizure impact, part of the observed associations may reflect shared measurement constructs rather than independent clinical effects. This may partly explain the large effect sizes observed for cognitive impairment. Future studies using more distinct or domain-specific measures would help to better disentangle these relationships. Finally, the continuous variables were broken down into categories for the analysis and evaluation of clinical significance of the findings, but this could result in a loss of statistical power of tests, and the relationships of the dose and effects may have been obscured. Moreover, some effect estimates were very large, which may indicate model instability or small numbers in certain categories. The association between polytherapy and QoL may be influenced by confounding by indication, as patients with more severe disease are more likely to receive combination therapy. This should be taken into account when interpreting the results.

## 5. Conclusions

In this study, reduced quality of life was highly prevalent among patients with epilepsy in Southern Kazakhstan and was strongly associated with both clinical and psychosocial factors. Cognitive impairment and poor medication adherence emerged as the most influential key correlates of reduced QoL. Importantly, greater seizure severity was associated with both adherence and QoL and influences their observed relationship, although causal pathways cannot be determined. These findings underscore the need for a comprehensive, patient-centered approach to epilepsy management that extends beyond seizure control alone.

## Figures and Tables

**Table 1:** Socio-demographic characteristics of patients by QoL.

	Total (*n* = 1100)	Quality of Life		*p*-Value
		Good QoL (*n* = 419; 38%)	Moderately or Severely Reduced QoL (*n* = 681; 62%)	
Age of onset (years), mean (SD)	27 (16)	25 (14)	28 (17)	<0.001
Gender, *n* (%)				0.086
Female	557 (51)	226 (54)	331 (49)	
Male	543 (49)	193 (46)	350 (51)	
Living area, *n* (%)				0.834
Urban area	540 (49)	204 (49)	336 (49)	
Rural area	560 (51)	215 (51)	345 (51)	
Education level, *n* (%)				<0.001
Low	64 (6)	8 (2)	56 (8)	
Medium	932 (85)	358 (85)	574 (84)	
High	104 (9)	53 (13)	51 (7)	
Marital status, *n* (%)				<0.001
Single	314 (28)	144 (34)	170 (25)	
Married	524 (48)	221 (53)	303 (44)	
Divorced	161 (15)	48 (12)	113 (17)	
Widower	99 (9)	6 (1)	93 (14)	
Disability, *n* (%)				<0.001
None	536 (49)	252 (60)	284 (42)	
Group I	40 (4)	7 (2)	33 (5)	
Group II	309 (28)	81 (19)	228 (33)	
Group III	214 (19)	79 (19)	135 (20)	
Income, *n* (%)				<0.001
<100 USD	332 (30)	157 (38)	175 (26)	
100–199 USD	290 (26)	98 (23)	192 (28)	
200–299 USD	329 (30)	91 (22)	238 (35)	
≥300 USD	148 (14)	73 (17)	75 (11)	

**Table 2:** Clinical characteristics by QoL.

	Total (*n* = 1100)	Quality of Life		*p*-Value
		Good QoL (*n* = 419; 38%)	Moderately or Severely Reduced QoL (*n* = 681; 62%)	
Duration of epilepsy (years), mean (SD)	14 (10)	11 (8)	15 (10)	<0.001
Family history of epilepsy, *n* (%)				0.304
No	600 (55)	217 (52)	283 (56)	
Yes	95 (8)	36 (8)	59 (9)	
Do not know	405 (37)	166 (40)	239 (35)	
Numbers of AED taken, *n* (%)				0.006
1 AED	916 (83)	364 (87)	552 (81)	
2 AEDs	176 (16)	55 (13)	121 (18)	
3 AEDs	8 (1)	0	8 (1)	
Frequency of taking AEDs, *n* (%)				0.007
One time	16 (1)	11 (3)	5 (1)	
Two times	954 (87)	369 (88)	585 (86)	
Three times	130 (12)	39 (9)	91 (13)	
Drug resistance, *n* (%)				0.017
Yes	182 (17)	55 (13)	127 (19)	
CT/MRI, *n* (%)				<0.001
Normal	197 (18)	98 (23)	99 (15)	
Structural abnormalities	889 (81)	314 (75)	575 (84)	
Not examined	14 (1)	7 (2)	7 (1)	
Concomitant chronic disease, *n* (%)				<0.001
Mental	46 (4)	5 (1)	41 (6)	
Neurological	545 (50)	190 (45)	355 (52)	
Somatic	149 (14)	48 (11)	101 (15)	

**Table 3:** Seizure-related characteristics by QoL.

	Total (*n* = 1100)	Quality of Life		*p*-Value
		Good QoL (*n* = 419; 38%)	Moderately or Severely Reduced QoL (*n* = 681; 62%)	
Etiology of epilepsy, *n* (%)				<0.001
Unknown	412 (37)	192 (46)	220 (32)	
Infectious	30 (3)	10 (2)	20 (3)	
Structural	658 (60)	217 (52)	441 (65)	
Seizure time, *n* (%)				0.228
Morning	211 (19)	85 (20)	126 (19)	
Afternoon	325 (30)	120 (29)	205 (30)	
Evening	329 (30)	122 (29)	207 (30)	
Before bed	61 (5)	31 (7)	30 (4)	
During sleep	174 (16)	61 (15)	113 (17)	
Aura, *n* (%)				<0.001
Somatosensory	288 (26)	102 (24)	186 (27)	
Visual	34 (3)	15 (4)	19 (3)	
Auditory	68 (6)	19 (5)	49 (7)	
Taste	27 (2)	12 (3)	15 (2)	
Olfactory	41 (4)	21 (5)	20 (3)	
Abdominal	182 (17)	57 (14)	125 (18)	
Emotional	77 (7)	25 (6)	52 (8)	
Mental	79 (7)	20 (5)	59 (9)	
Motor	43 (4)	16 (4)	27 (4)	
Type of seizures, *n* (%)				<0.001
Simple partial	137 (12)	78 (19)	59 (9)	
Complex partial	501 (46)	214 (51)	287 (42)	
Secondary generalized	228 (21)	44 (10)	184 (27)	
Generalized tonic-clonic	234 (21)	83 (20)	151 (22)	
Seizure frequency, *n* (%)				<0.001
1 seizure per year	51 (5)	33 (8)	18 (3)	
3–10 seizures per year	233 (21)	124 (29)	109 (16)	
1–3 seizures per month	232 (21)	96 (23)	136 (20)	
1 or more seizures per week	584 (53)	166 (40)	418 (61)	
Postictal confusion [yes], *n* (%)	757 (69)	232 (55)	525 (77)	<0.001
Serial seizures [yes], *n* (%)	638 (58)	180 (43)	458 (67)	<0.001
Provoking factors, *n* (%)				0.003
Reflex	351 (32)	161 (38)	190 (28)	
Menstrual cycle	13 (1)	8 (2)	5 (1)	
Alcohol consumption	118 (11)	37 (9)	81 (12)	
Treatment withdrawal	144 (13)	48 (11)	96 (14)	
Lack of sleep	192 (17)	64 (16)	128 (19)	
Emotional	171 (16)	65 (15)	106 (16)	
Meteorological factors	50 (4.5)	12 (3)	38 (5)	
Over-fatigue	61 (5.5)	24 (6)	37 (5)	
Post-seizure state, *n* (%)				<0.001
No changes	152 (14)	86 (21)	66 (10)	
Sleep	226 (20)	65 (15)	161 (24)	
Headaches	385 (35)	149 (36)	236 (35)	
Nausea, vomiting	131 (12)	38 (9)	93 (13)	
Todd’s paralysis	63 (6)	31 (7)	32 (5)	
Stupor	143 (13)	50 (12)	93 (13)	
Neurological status, *n* (%)				<0.001
Normal	479 (43)	261 (62)	218 (32)	
Micro symptomatology	492 (45)	141 (34)	351 (52)	
Gross focal neurological symptoms	129 (12)	17 (4)	112 (16)	

**Table 4:** Medication adherence, seizure severity, cognitive function, and stigma by quality-of-life status.

	Total (*n* = 1100)	Quality of Life		*p*-Value
		Good QoL (*n* = 419; 38%)	Moderately or Severely Reduced QoL (*n* = 681; 62%)	
MMAS, *n* (%)				<0.001
Low	830 (75)	245 (58)	585 (86)	
Medium	268 (24)	172 (41)	96 (14)	
High	2 (1)	2 (1)	0	
LSSS, *n* (%)				0.004
Mild seizures	215 (19)	102 (24)	113 (17)	
Moderate seizures	878 (80)	316 (75)	562 (82)	
Severe seizures	7 (1)	1 (1)	6 (1)	
MoCA, *n* (%)				<0.001
Mild cognitive impairment	898 (82)	375 (89)	523 (77)	
Moderate or severe cognitive impairment	156 (14)	2 (1)	154 (22)	
Normal	46 (4)	42 (10)	4 (1)	
ESS, *n* (%)				<0.001
Average	720 (66)	273 (65)	447 (66)	
High	234 (21)	10 (5)	214 (31)	
Low	146 (13)	126 (30)	20 (3)	

## Data Availability

The data supporting the findings of this study are available from the corresponding author upon reasonable request.

## Supplementary Materials

The following supporting information can be downloaded at: https://www.mdpi.com/article/10.3390/jcm15093311/s1, Table S1. Logistic regression model for seizure severity predictors; Table S2. Logistic regression model for reduced QoL with continuous MoCA, MMAS and LSSS scores.

## Abbreviations

The following abbreviations are used in this manuscript:

ASM	Antiseizure medication
QoL	Quality of Life
CT	Computed tomography
MRI	magnetic resonance imaging
QOLIE	Quality of Life in Epilepsy
MMAS	Morisky Medication Adherence Scale
LSSS	Liverpool Seizure Severity Scale
MoCA	Montreal Cognitive Assessment
ESS	Epilepsy Stigma Scale
SD	standard deviation
OR	odds ratio

## References

[1] Hohmann L., Berger J., Kastell S.U., Holtkamp M. (2023). Subjective cognition is linked to everyday functioning in epilepsy. Epilepsia Open.

[2] Novak A., Vizjak K., Rakusa M. (2022). Cognitive impairment in people with epilepsy. J. Clin. Med..

[3] Busch R.M., Dalton J.E., Jehi L., Ferguson L., Krieger N.I., Struck A.F., Hermann B.P. (2023). Association of neighborhood deprivation with cognitive and mood outcomes in adults with pharmacoresistant temporal lobe epilepsy. Neurology.

[4] Ehrlich T., Reyes A., Paul B.M., Uttarwar V., Hartman S., Mathur K., Chang Y.-H.A., Hegde M., Shih J.J., McDonald C.R. (2019). Beyond depression: The impact of executive functioning on quality of life in patients with temporal lobe epilepsy. Epilepsy Res..

[5] Masoudian N., Moradpour M., Samaei A., Ehsani F., Ziari A. (2020). Assessment of cognitive functions and related risk factors in Iranian patients with generalized epilepsy as compared to patients with non-epileptic neurological disorders. Curr. J. Neurol..

[6] Giovagnoli A.R., Parente A., Tarallo A., Casazza M., Franceschetti S., Avanzini G. (2014). Self-rated and assessed cognitive functions in epilepsy: Impact on quality of life. Epilepsy Res..

[7] Lin H., Holmes G.L., Kubie J.L., Muller R.U. (2009). Recurrent seizures induce a reversible impairment in a spatial hidden goal task. Hippocampus.

[8] Rudzinski L.A., Meador K.J. (2013). Epilepsy and neuropsychological comorbidities. Contin. Lifelong Learn. Neurol..

[9] Quon R.J., Mazanec M.T., Schmidt S.S., Andrew A.S., Roth R.M., MacKenzie T.A., Sajatovic M., Spruill T., Jobst B.C. (2020). Antiepileptic drug effects on subjective and objective cognition. Epilepsy Behav..

[10] Shafiyev J., Karadaş Ö. (2024). The assessment of the impact of antiepileptic drugs on cognitive functions via N-200/P-300 potentials and neuropsychological measures. Neurol. Sci..

[11] Sen A., Newton C.R., Ngwende G. (2025). Epilepsy in low- to middle-income countries. Curr. Opin. Neurol..

[12] Zhang X., Wang Z., Xiang F., Li Y., Shi X., Shao C., Lang S., Wang X. (2025). Etiologies of patients with adult-onset epilepsy over the past 25 years: A retrospective study in China. BMC Neurol..

[13] Kek-Laflamme A., Schaper F.L., Whittingstall K., Pennell P.B., Young G.S., Marshall G.A., Larivière S., Sarkis R.A. (2026). Brain Structural Changes and Cognitive-Clinical Profiles in Late-Onset Unexplained Epilepsy. Neurology.

[14] Eberhart T., Kämmer J., Ellßel C., Flemming D., Pelizäus H. (2025). Problems and needs in everyday life of people with late-onset epilepsy: A scoping review categorization using the international classification of functioning, disability and health (ICF). Seizure Eur. J. Epilepsy.

[15] Hoxhaj P., Habiya S.K., Sayabugari R., Balaji R., Xavier R., Ahmad A., Khanam M., Kachhadia M.P., Patel T., Abdin Z.U. (2023). Investigating the impact of epilepsy on cognitive function: A narrative review. Cureus.

[16] Paramonova A.I., Lysova K.D., Timechko E.E., Senchenko G.V., Sapronova M.R., Dmitrenko D.V. (2024). Cognitive impairment in childhood-onset epilepsy. Epilepsy Paroxysmal Cond..

[17] Awan S.A., Khawaja I., Babar M., Khan F. (2022). Prevalence of non-adherence to antiepileptic drugs in patients with epilepsy presenting to emergency with fits. Cureus.

[18] Donahue M.A., Akram H., Brooks J.D., Modi A.C., Veach J., Kukla A., Benard S.W., Herman S.T., Farrell K., Ficker D.M. (2025). Barriers to medication adherence in people living with epilepsy. Neurol. Clin. Pract..

[19] Wang H., Liu X. (2025). Medication self-management in patients with epilepsy: A narrative review of current status, influencing factors, and intervention strategies. Front. Neurol..

[20] Kim Y., Jang Y. (2026). A narrative review of antiseizure medication adherence in epilepsy: Determinants to clinical implications. Encephalitis.

[21] Al-Aqeel S., Gershuni O., Al-Sabhan J., Hiligsmann M. (2020). Strategies for improving adherence to antiepileptic drug treatment in people with epilepsy. Cochrane Database Syst. Rev..

[22] Narodova E.A. (2025). Digital Tools for Seizure Monitoring and Self-Management in Epilepsy: A Narrative Review. J. Clin. Med..

[23] Mbuba C.K., Newton C.R. (2009). Packages of care for epilepsy in low-and middle-income countries. PLoS Med..

[24] Tabyshova A., Sooronbaev T., Akylbekov A., Mademilov M., Isakova A., Erkinbaeva A., Magdieva K., Chavannes N.H., Postma M.J., van Boven J.F.M. (2022). Medication availability and economic barriers to adherence in asthma and COPD patients in low-resource settings. npj Prim. Care Respir. Med..

[25] Rocamora R., Chavarría B., Pérez E., Pérez-Enríquez C., Barguilla A., Panadés-de Oliveira L., Principe A., Zucca R. (2021). Mood disturbances, anxiety, and impact on quality of life in patients admitted to epilepsy monitoring units. Front. Neurol..

[26] Kaddumukasa M., Mugenyi L., Lhatoo S., Sewankambo N., Blixen C., Sajatovic M., Katabira E. (2019). Seizure severity is associated with poor quality of life in people living with epilepsy (PLWE) in Uganda: A cross-sectional study. Epilepsy Behav..

[27] van Hezik-Wester V., de Groot S., Kanters T., Versteegh M., Wagner L., Ardesch J., Brouwer W., van Exel J. (2022). Burden of illness in people with medically refractory epilepsy who suffer from daily to weekly seizures: 12-month follow-up of participants in the EPISODE study. Front. Neurol..

[28] Hovinga C.A., Asato M.R., Manjunath R., Wheless J.W., Phelps S.J., Sheth R.D., Pina-Garza J.E., Zingaro W.M., Haskins L.S. (2008). Association of non-adherence to antiepileptic drugs and seizures, quality of life, and productivity: Survey of patients with epilepsy and physicians. Epilepsy Behav..

[29] Shiek Ahmad B., Hill K.D., O’Brien T.J., Gorelik A., Habib N., Wark J.D. (2012). Falls and fractures in patients chronically treated with antiepileptic drugs. Neurology.

[30] Zafar A., Shahid R., Nazish S., Aljaafari D., Alkhamis F.A., Alsalman S., Msmar A.H., Abbasi B., Alsulaiman A.A., Alabdali M. (2019). Nonadherence to antiepileptic medications: Still a major issue to be addressed in the management of epilepsy. J. Neurosci. Rural. Pract..

[31] Alqwaifly M. (2020). Nonadherence to antiepileptic medications: A cross sectional study in Saudi Arabia. Int. J. Med. Dev. Ctries..

[32] Verma R., Gupta R., Shafqat N., Phalswal U. (2024). A study to assess medication adherence and quality of life among epilepsy patients seeking treatment at AIIMS Bhopal. J. Fam. Med. Prim. Care.

[33] Hamedi-Shahraki S., Eshraghian M.-R., Yekaninejad M.-S., Nikoobakht M., Rasekhi A., Chen H., Pakpour A. (2019). Health-related quality of life and medication adherence in elderly patients with epilepsy. Neurol. Neurochir. Pol..

[34] Walker M.C., Galovic M., Álvarez-Barón E., Strzelczyk A. (2025). Seizures beget more than seizures: Understanding the cellular, structural, individual and societal impact of seizures in epilepsy. Epilepsia Open.

[35] Viteva E.I. (2014). Seizure frequency and severity: How really important are they for the quality of life of patients with refractory epilepsy. Ann. Indian Acad. Neurol..

[36] Englot D.J. (2023). Networks inhibited and networks excited: Loss of consciousness in epilepsy. Epilepsy Curr..

[37] Vingerhoets G. (2006). Cognitive effects of seizures. Seizure.

[38] Hohmann L., Berger J., Kastell S.-U., Holtkamp M. (2022). Perceived epilepsy-related stigma is linked to the socioeconomic status of the residence. Front. Public Health.

[39] Blixen C., Ogede D., Briggs F., Aebi M.E., Burant C., Wilson B., Terashima J.P., Sajatovic M. (2020). Correlates of stigma in people with epilepsy. J. Clin. Neurol..

[40] Malik N.I., Fatima R., Ullah I., Atta M., Awan A., Nashwan A.J., Ahmed S. (2022). Perceived stigma, discrimination and psychological problems among patients with epilepsy. Front. Psychiatry.

