# Supplementary material for: Medication Adherence and Quality of Life in Epilepsy: The Potential Role of Seizure Severity in the Association Between Them
Source: J Clin Med. 2026 Apr 27;15(9):3311. doi: 10.3390/jcm15093311 (PMC13164267; doi:10.3390/jcm15093311)
Supplement: Supplementary file 1 [file jcm-15-03311-s001.zip › jcm-4280225-supplementary.pdf]

Supplementary Table S1. Logistic regression model for seizure severity predictors

	Unadjusted model		Adjusted model	
	OR [95% CI]	*p*-value	OR [95% CI]	*p*-value
Age of onset	0.98 [0.97-0.99]	<0.001	0.98 [0.97-0.99]	<0.001
Gender [ref.female]				
Male	1.15 [0.86-1.55]	0.351	1.08 [0.79-1.47]	0.629
Education level [ref. high]				
Medium	0.58 [0.32-1.04]	0.068	0.52 [0.28-0.95]	0.032
Low	2.33 [0.73-7.43]	0.152	1.45 [0.43-4.86]	0.548
Duration of epilepsy	1.01 [0.99-1.02]	0.394	0.98 [0.97-1.00]	0.119
Number of AEDs [ref. 1]				
2 or more	1.07 [1.01-1.17]	0.007	6.18 [0.43 -88.4]	0.180
Drug resistance [ref. no]	1.13 [0.71-1.61]	0.748	0.15 [0.01-2.15]	0.163
MoCA [ref. normal]				
Mild cognitive impairment	0.36 [0.13-1.03]	0.057	0.40 [0.14-1.16]	0.092
Moderate or severe cognitive impairment	0.49 [0.16-1.52]	0.220	0.59 [0.19-1.93]	0.391
MRI/CT [ref. normal]				
Structural abnormalities	1.44 [1.12-1.94]	0.015	1.61[1.09-2.36]	0.016
MMAS [ref. medium and high]				
Low	1.17 [1.02-1.43]	0.036	1.18 [1.01-1.48]	0.002

Supplementary Table S2. Logistic regression model for reduced QoL with continuous MoCA, MMAS and LSSS scores

	Unadjusted model		Model 1		Model 2	
	OR [95% CI]	*p*-value	OR [95% CI]	*p*-value	OR [95% CI]	*p*-value
Age of onset	1.02 [1.01-1.02]	<0.001	1.02 [1.01-1.04]	<0.001	1.03 [1.01-1.04]	<0.001
Gender [ref.female]						
Male	1.24 [0.97-1.58]	0.086	0.95 [0.69-1.31]	0.574	0.95 [0.69-1.31]	0.742
Education level [ref. high]						
Medium	1.67 [1.11-2.50]	0.014	1.60 [0.90-2.84]	0.107	1.68 [0.94-3.01]	0.078
Low	7.27 [3.16-16.8]	<0.001	4.51 [1.45-13.9]	0.009	4.30 [1.39-13.3]	0.011
Duration of epilepsy	1.05 [1.03-1.06]	<0.001	1.03 [1.01-1.06]	0.002	1.04 [1.01-1.06]	0.001
Number of AEDs [ref. 1]						
2 or more	1.45 [1.03-2.05]	0.035	1.71 [0.26-11.3]	0.576	1.63 [0.25-10.8]	0.610
Drug resistance [ref. no]	1.52 [1.08-2.14]	0.017	0.70 [0.114.68]	0.715	0.76 [0.11-5.04]	0.775
MoCA score	0.56 [0.52-0.61]	<0.001	0.63 [0.58-0.68]	<0.001	0.63 [0.58-0.68]	<0.001
MRI/CT [ref. normal]						
Structural abnirmalities	1.81 [1.33-2.47]	<0.001	1.37 [0.91-2.07]	0.131	1.38 [0.92-2.10]	0.121

MMAS score	0.43 [0.37-0.49]	<0.001	0.54 [0.46-0.64]	<0.001	0.55 [0.46-0.65]	<0.001
LSSS score	1.03 [1.01-1.05]	0.027			1.04 [1.01-1.08]	0.011
